# Expression and function of visfatin (Nampt), an adipokine-enzyme involved in inflammatory pathways of osteoarthritis

**DOI:** 10.1186/ar4467

**Published:** 2014-01-31

**Authors:** Marie-Charlotte Laiguillon, Xavier Houard, Carole Bougault, Marjolaine Gosset, Geoffroy Nourissat, Alain Sautet, Claire Jacques, Francis Berenbaum, Jérémie Sellam

**Affiliations:** 1 INSERM UMRS_938, UPMC, Univ Paris 06, 184 rue du Faubourg Saint-Antoine, 75012 Paris, France; 2 EA 2496, Paris Descartes University, 1 rue Maurice Arnoux, 92120 Montrouge, France; 3 Department of Orthopaedic Surgery and Traumatology, Saint-Antoine Hospital, AP-HP, Univ Paris 06, 184 rue du Faubourg Saint-Antoine, 75012 Paris, France; 4 Department of Rheumatology, Assistance Publique – Hôpitaux de Paris, Saint-Antoine Hospital, 184 rue du Faubourg Saint-Antoine, 75012 Paris, France; 5 Inflammation–Immunopathology–Biotherapy Department (DHU i2B), 184 rue du Faubourg Saint-Antoine, 75012 Paris, France

## Abstract

**Introduction:**

Visfatin is an adipokine that may be involved in intertissular joint communication in osteoarthritis (OA). With a homodimeric conformation, it exerts nicotinamide phosphoribosyltransferase (Nampt) enzymatic activity, essential for nicotinamide adenine dinucleotide biosynthesis. We examined the tissular origin and conformation of visfatin/Nampt in human OA joints and investigated the role of visfatin/Nampt in chondrocytes and osteoblasts by studying Nampt enzymatic activity.

**Methods:**

Synovium, cartilage and subchondral bone from human OA joints were used for protein extraction or incubated for 24 hours in serum-free media (conditioned media), and synovial fluid was obtained from OA patients. Visfatin/Nampt expression in tissular extracts and conditioned media was evaluated by western blot and enzyme-linked immunosorbent assay (ELISA), respectively. Nampt activity was assessed in OA synovium by colorimetric assay. Primary cultures of murine chondrocytes and osteoblasts were stimulated with visfatin/Nampt and pretreated or not with APO866, a pharmacologic inhibitor of Nampt activity. The effect on cytokines, chemokines, growth factors and hypertrophic markers expression was examined by quantitative reverse transcriptase polymerase chain reaction and/or ELISA.

**Results:**

In tissular explants, conditioned media and synovial fluid, visfatin/Nampt was found as a homodimer, corresponding to the enzymatically active conformation. All human OA joint tissues released visfatin/Nampt (synovium: 628 ± 106 ng/g tissue; subchondral bone: 195 ± 26 ng/g tissue; cartilage: 152 ± 46 ng/g tissue), with significantly higher level for synovium (*P* <0.0005). Nampt activity was identified *ex vivo* in synovium. *In vitro*, visfatin/Nampt significantly induced the expression of interleukin 6, keratinocyte chemoattractant and monocyte chemoattractant protein 1 in chondrocytes and osteoblasts. APO866 decreased the mRNA and protein levels of these pro-inflammatory cytokines in the two cell types (up to 94% and 63% inhibition, respectively). Levels of growth factors (vascular endothelial growth factor, transforming growth factor β) and hypertrophic genes were unchanged with treatment.

**Conclusion:**

Visfatin/Nampt is released by all human OA tissues in a dimeric enzymatically active conformation and mostly by the synovium, which displays Nampt activity. The Nampt activity of visfatin is involved in chondrocyte and osteoblast activation, so targeting this enzymatic activity to disrupt joint tissue interactions may be novel in OA therapy.

## Introduction

Osteoarthritis (OA) is a chronic joint disease characterized by cartilage breakdown, bone remodeling, osteophyte development and synovium inflammation [1]. The synovial membrane, which contains metabolically highly active cells (that is, synoviocytes), is physiologically important because it both nourishes chondrocytes via the synovial fluid and joint space and removes metabolites and products of matrix degradation [2]. In OA, synovium is inflamed and characterized by hypertrophic and hyperplasic synoviocytes and infiltrating mononuclear cells. All of these cells produce interleukin (IL)-1β, IL-6, IL-8 and tumor necrosis factor alpha (TNFα), major proinflammatory cytokines in OA [3,4]. This cytokinic environment results in activated chondrocytes and subchondral osteoblasts that release prodegradative enzymes responsible for joint disruption as well as proinflammatory cytokines and chemokines such as IL-6, monocyte chemoattractant protein 1 (MCP-1), IL-8 or TNFα, thus perpetuating a vicious inflammatory circle.

Recent data support a direct communication between the subchondral bone and cartilage via a process of diffusion through vessels, microcracks and fissures [5]. This diffusion permits the exchange of soluble products with the ability to modulate the activities of resident cells in these tissues [6,7]. OA synovium may also be involved in this pathological tissular network because it synthesizes synovial fluid, releasing proinflammatory and prodegradative mediators participating in joint disruption. As emphasized by Loeser and colleagues, we need to address which of the factors released from synovium promote cartilage degradation and bone remodeling [1].

Among the soluble mediators released by synovium potentially involved in OA pathophysiology, the so-called adipokines, known as mediators mainly from adipose tissue and found in biological fluids, may participate in synovium–bone and synovium–cartilage interactions [8-10]. Adipokines have pleiotropic effects and participate in several metabolic, immune and inflammatory processes. They contribute strikingly to the low-grade inflammatory state observed in obese subjects and thus to the pathophysiologic aspects of metabolic diseases as well as some cancers. Among the adipokines, leptin and adiponectin have been extensively studied in OA [11] and may be crucial actors in the pathophysiologic features of the metabolic OA phenotype [12].

Interest is growing in the adipokine visfatin, also called pre-B-cell colony-enhancing factor [13] or nicotinamide phosphoribosyltransferase (Nampt) [14]. This 52 kDa protein is constitutively synthesized by adipose tissue but also by many other tissues, including synovium and cartilage [15-17] and peripheral blood mononuclear cells [18], which raises the issue of its strict definition as an adipokine. Considering its various names, visfatin/Nampt/pre-B-cell colony-enhancing factor is a complex adipokine initially discovered as a molecule secreted by activated lymphocytes in bone marrow and able to stimulate the formation of pre-B cells [13]. Visfatin also acts as a proinflammatory cytokine able to induce TNFα, IL-6 and IL-1β [14,18].

Interestingly, visfatin is considered an adipokine-enzyme with the name Nampt because it has Nampt enzymatic activity due to a homodimeric conformation creating the enzymatic active site, according to crystallographic structure study [19]. Visfatin/Nampt is involved in the biosynthetic pathway of nicotinamide adenine dinucleotide (NAD) by converting nicotinamide into nicotinamide mononucleotide, and represents the limiting factor of this enzymatic reaction. NAD is an essential cofactor for many intracellular processes: it allows the transfer of electrons in redox reactions, modulates the activity of key regulators in cell longevity and acts as a cofactor in DNA repair or histone deacetylation [14,20]. This enzymatic activity can be inhibited by a pharmacologic competitive inhibitor, APO866 (also known as FK866 or WK175), which binds to the active site formed by the dimer [21]. This inhibitor greatly decreases the concentration of intracellular NAD, thus resulting in apoptosis of tumoral cells in many cancer types [22,23]. In contrast, in non-excess proliferating cells, such as human monocytes, APO866 reduces the production of inflammatory cytokines without affecting their viability [24].

In rheumatoid arthritis, visfatin/Nampt is elevated in plasma of patients [25] and may participate in the inflammatory process by orchestrating fibroblast motility and by promoting cytokine synthesis [16,26]. Visfatin/Nampt blockade with APO866 can prevent or limit joint destruction and inflammation in collagen-induced arthritis [24,27]. Conversely, little is known about visfatin/Nampt in OA or its effects in chondrocytes and osteoblasts. We previously showed that visfatin/Nampt is produced by IL-1β-stimulated OA chondrocytes and may induce a prodegradative and proinflammatory phenotype of chondrocytes characterized by the induction of matrix metalloproteinase (MMP)-3 and MMP-13 and synthesis of prostaglandin E_2_[17]. These activities could be mediated in part by the insulin receptor signaling pathways, as a recent study also showed an inhibition of the production of proteoglycan induced by visfatin/Nampt via this pathway [17,28,29]. However, the expression and conformation of visfatin/Nampt within the OA joint and the involvement of the enzymatic activity in visfatin/Nampt-stimulated chondrocytes are poorly known. Furthermore, the responsiveness of osteoblasts to visfatin/Nampt still remains unknown.

In this study, we aimed to address the expression, conformation and enzymatic properties of visfatin/Nampt in human OA joints, to decipher the proinflammatory role of this adipokine in two cell types involved in OA (that is, chondrocytes and osteoblasts), and to connect the cytokinic and enzymatic effects of this adipokine enzyme, investigating whether these effects are mediated by Nampt activity.

## Methods

### Materials

All reagents were purchased from Sigma-Aldrich (Lyon, France), unless stated otherwise. The human visfatin/Nampt enzyme-linked immunosorbent assay (ELISA) kit was from Adipogen (San Diego, CA, USA). New-born Swiss mice were from Janvier (St Berthevin, France). The anti-human visfatin/Nampt polyclonal antibody was from Alexis (Paris, France). The immunoblot nitrocellulose transfer membranes for western blot analysis were from Whatman (Dassel, Germany). The western blot enhanced chemiluminescence system and kaleidoscope prestained standards were from Bio-Rad (Marnes-la-Coquette, France). The Cyclex visfatin/Nampt colorimetric assay kit was from MBL International (Woburn, MA, USA). Recombinant mouse and human visfatin/Nampt (produced in *Escherichia coli* with residual lipopolysaccharide contamination <100 pg/ml according to the manufacturer) was from Alexis Biochemicals (Paris, France). APO866, a gift from Astellas Pharma (Munich, Germany), was provided by Alexander So (Rheumatology Department, Centre Hospitalier Universitaire Vaudois and University of Lausanne, Switzerland) and also purchased from Alexis Biochemicals. IL-1β was from PeproTech (Rocky Hill, NJ, USA).

### Collection of osteoarthritis human material

Human OA knee explants and synovial fluids were obtained from patients undergoing total joint replacement surgery for OA at Saint-Antoine Hospital (Paris, France). Informed consent for use of tissue was obtained from each patient before surgery. The diagnosis of OA was based on clinical and radiographic evaluations according to the criteria of the American College of Rheumatology [30]. All crude tissular explants were manually dissected to obtain separate samples of each tissue type (that is, cartilage, synovial membrane and subchondral bone).

The explants were cut into small pieces (~1 mm^3^), washed several times with phosphate-buffered saline and incubated in RPMI-1640 culture medium supplemented with 100 U/ml penicillin, 100 μg/ml streptomycin, and 4 mM glutamine for 24 hours at 37°C. Conditioned media (CM) were then collected, centrifuged (3,000 × *g* for 5 minutes) and stored at −80°C. Each volume of medium was normalized to wet weight of explants (6 ml/g tissue), as described previously [31].

In parallel, explants of each tissue type were frozen and ground under liquid nitrogen using a pestle and mortar. Protein was then extracted with lysing buffer for western blot experiments. Experiments using human samples have been approved by a French Institutional Review Board (Comité de Protection des Personnes Ile de France V).

### Primary culture of murine articular chondrocytes

Mouse primary chondrocytes were isolated from articular cartilage of 5-day-old to 6-day-old newborn Swiss mice as described elsewhere [32]. After 1 week of amplification, cells were incubated in serum-free Dulbecco’s modified Eagle’s medium (DMEM) containing 0.1% of bovine serum albumin for 24 hours before treatment.

### Primary culture of murine osteoblasts

As described previously [33], mouse primary osteoblasts were isolated from calvaria of 5-day-old to 6-day-old newborn Swiss mice; the calvaria phenotype is considered close to that of subchondral bone [34]. Osteoblasts were cultured for 2 weeks in DMEM/HAM-F12 supplemented with 100 U/ml penicillin, 100 μg/ml streptomycin, and 4 mM glutamine. In the first week, cells were grown in DMEM/HAM-F12-PS-Glu enriched with 10% serum and vitamin C (50 μg/ml). In the second week, β-glycerol phosphate (5 mM) was added to the same culture medium. Before treatment, cells were weaned for 24 hours in a serum-free medium, DMEM/HAM-F12-PS-Glu and 0.1% bovine serum albumin, and treatments involved use of this same medium.

All experiments with murine articular chondrocytes and osteoblasts were performed according to the protocols approved by French and European ethics committees (Comité Régional d’Ethique en Expérimentation Animale N°3 de la région Ile de France).

### *Ex vivo* assessment of human visfatin/Nampt by western blot

Western blots were performed on protein extracts from crude tissular explants, synovial fluids and CM. Crude tissular explants were lysed in a buffer containing 20 mM Tris–HCl (pH 7.6), 120 mM NaCl, 10 mM ethylenediamine tetraacetic acid pH 8, 10% glycerol, 1% Nonidet P40, 10 mM sodium pyrophosphate and 1 protease inhibitor cocktail (Roche Diagnostics, Indianapolis, IN, USA). Proteins of all samples (45 μg tissular extracts, equal volume of CM reported to the tissue mass and 50 μg synovial fluids) were then separated on Criterion XT 4 to 12% Bis–Tris Gel (Bio-Rad) and transferred to nitrocellulose membranes. Monomeric and polymeric proteins were detected by immunoblotting using specific polyclonal antibody (1/2,000) or a monoclonal antibody (1/2,000) against human visfatin/Nampt (Enzo, Villeurbanne, France). Amplification of the signal was obtained using a secondary rabbit-horseradish peroxidase (1/1,000) antibody anti-human IgG (Paris Anticorps, Compiègne, France). Recombinant human visfatin/Nampt was used as positive control. The control of proteins deposition was performed by quantification of total protein of each sample using the Bio-Rad protein assay kit (Bio-Rad, Munich, Germany) and staining of actin using an anti-actin antibody (1/,2000; Sigma-Aldrich, Lyon, France) (data not shown).

The monomeric form of visfatin/Nampt (that is, enzymatically inactive conformation) was investigated in denaturing conditions and the polymeric form (that is, enzymatically active conformation) was investigated in nondenaturing conditions (without β-mercaptoethanol). Results were revealed using an Immun-Star WesternC Chemiluminescence Kit (Bio-Rad) and pictures were obtained by MultiGauge version 3.0 (Fujifilm, Bois d’Arcy, France).

### Measurement of visfatin/Nampt enzymatic activity

To quantify the enzymatic activity of visfatin/Nampt in OA human synovium, protein extracts from crude tissue of three patients were assayed using the Cyclex Nampt Colorimetric Assay Kit (MBL International). This assay measures the kinetics of the production of NAD, the final product of the visfatin/Nampt pathway. During this assay, all components being in a saturated condition, the only variation observed is exclusively linked to the concentration of visfatin/Nampt in the tissue extracts. Absorbance of the derived product was read at 450 nm using a spectrophotometer. In order to confirm the specificity of the assay, pretreatment by the specific inhibitor of Nampt, APO866 (10 nM), was made by incubating the recombinant visfatin/Nampt and two other synovium samples for 1 hour at 37°C. The curves were then drawn for the absorbance (optical density) over time (minutes). The initial Nampt enzymatic activity of each sample was thus calculated as the slope of the curve and definitive results were given per minute.

### Treatment of primary cultures of chondrocytes and osteoblasts

Confluent chondrocytes and osteoblasts were stimulated with recombinant visfatin/Nampt (20, 50 and 100 nM) in serum-free medium for 24 hours. To assess Nampt enzymatic activity, cells were pretreated for 4 hours with the Nampt inhibitor APO866 (10 nM) before the addition of visfatin/Nampt. The effect of 10 nM APO866 on chondrocytes was considered efficient after a dose–effect experiment under visfatin/Nampt stimulation (data not shown) and previous results [28]. To determine the optimal concentration of APO866 for osteoblasts, cells underwent dose–effect experiments with 1, 10 and 100 nM APO866. The cytotoxic effects of APO866 on cells were assayed using the Cytotoxicity Detection Kit (lactate dehydrogenase; Roche, Mannheim, Germany).

### RNA extraction and quantitative real-time reverse transcriptase-polymerase chain reaction

Total RNA was extracted from chondrocytes and osteoblasts using the RNeasy kit (Qiagen, Courtaboeuf, France) and concentrations were determined using a spectrophotometer (Eppendorf, Le Pecq, France). Reverse transcription involved 500 ng total RNA with the Omniscript RT kit (Qiagen). mRNA levels of IL-6, keratinocyte chemoattractant (Kc; the murine equivalent of IL-8), MCP-1, vascular endothelial growth factor, transforming growth factor beta, runt-related transcription factor 2, type X collagen and Indian hedgehog were quantified using a Light Cycler LC480 (Roche Diagnostics) as described previously [35]. Levels of mRNA were normalized to those of murine hypoxanthine guanine phosphoribosyltransferase. Specific mouse primer sequences are referenced in Table S1 in Additional file 1.

### ELISA assessment of visfatin/Nampt, IL-6, keratinocyte chemoattractant and MCP-1 levels

The protein concentration of visfatin/Nampt released by all OA joint tissues in CM was measured by ELISA kit (AdipoGen, Liestal, Switzerland). The limit of detection was 30 pg/ml. IL-6, Kc and MCP-1 concentrations were measured in CM using the Quantikine ELISA kit (R&D Systems, Lille, France). The limits of detection were 1.6, 2.0 and 2.0 pg/ml, respectively. The values were averages of duplicate or triplicate tests.

### Statistical analysis

All data are reported as mean ± standard error of the mean. The Mann–Whitney test was used for analysis of visfatin/Nampt in synovium and other tissues, and the Wilcoxon test for analysis of the effect of visfatin/Nampt and APO866 on chondrocytes and osteoblasts. Analyses involved use of GraphPad Prism5 (GraphPad Software, San Diego, CA, USA). *P* ≤ 0.05 was considered statistically significant.

## Results

### Visfatin/Nampt is mainly produced by OA synovium in the human OA joint and is found in its enzymatically active conformation

**Figure 1:**
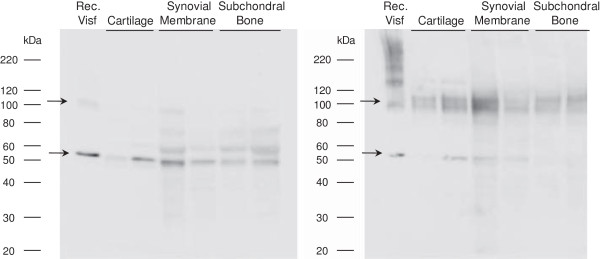
**Intratissular expression of visfatin/Nampt from osteoarthritic human joint tissues.** Human osteoarthritic joint tissues were obtained after surgery, separated, frozen and ground to obtain protein extracts. Western blot analysis of the visfatin/nicotinamide phosphoribosyltransferase (Nampt) protein level in tissues. Left panel: denaturing conditions, showing a monomeric conformation. Right panel: nondenaturing conditions, showing polymeric conformation. Arrows show bands specific to visfatin/Nampt.

We examined the presence of visfatin/Nampt within human OA tissues and investigated whether tissular visfatin/Nampt is found *ex vivo* in the dimeric conformation, essential for its enzymatic activity. Visfatin/Nampt was identified in all tissues and migrated as a 52 kDa band under denaturing conditions (Figure 1), which corresponds to the molecular weight of the visfatin/Nampt monomer. Under nondenaturing conditions, the 52 kDa band intensity decreased and a major band appeared at about 120 kDa due to visfatin/Nampt dimerization. Moreover, recombinant pure visfatin/Nampt showed a similar electrophoretic pattern. Similar results were obtained using a monoclonal antibody against human visfatin/Nampt confirming the specificity of the staining (data not shown).

**Figure 2:**
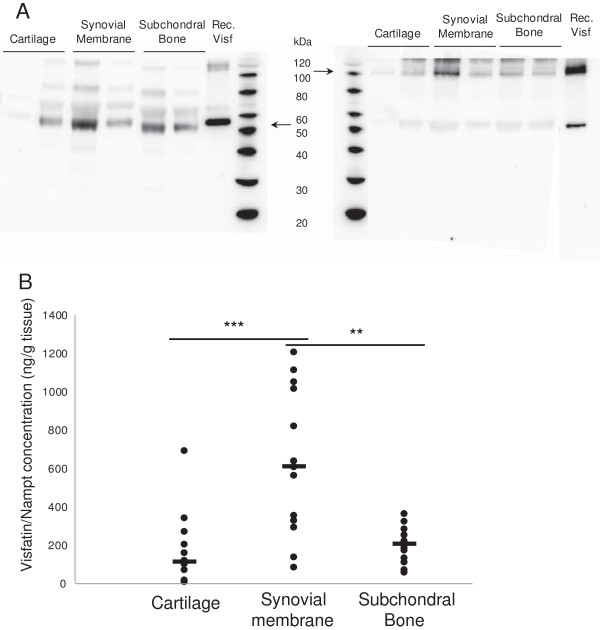
**Production of visfatin/Nampt by human osteoarthritic joint tissues.** Human osteoarthritic joint tissues were incubated for 24 hours in serum-free medium (6 ml/g tissue). Retrieved media were considered conditioned media. **(A)** Western blot analysis of visfatin/nicotinamide phosphoribosyltransferase (Nampt) protein level in conditioned media. Left panel: denaturing conditions, showing monomeric conformation. Right panel: nondenaturing conditions, showing dimeric conformation. Arrows show bands specific to visfatin/Nampt. **(B)** Enzyme-linked immunosorbent assay of visfatin/Nampt released by cartilage (*n* = 15), synovial membrane (*n* = 13) and subchondral bone (*n* = 13). **P < 0.001, ***P < 0.0005. Horizontal lines are medians. Each dot represents one sample.

We next assessed the production of visfatin/Nampt in CM by different OA tissues, first to determine the secreted form of visfatin/Nampt (under denaturing and nondenaturing conditions) and then visfatin/Nampt release. Western blot analysis under denaturing conditions revealed the production of visfatin/Nampt by all OA human joint tissues. Nondenaturing conditions allowed identification of the dimeric conformation of the protein, corresponding to its enzymatically active form (Figure 2A). We have performed quantification of visfatin/Nampt in CM using ELISA and have found that visfatin/Nampt was indeed released by all OA tissues (synovium, 628 ± 106 ng/g tissue; subchondral bone, 195 ± 26 ng/g tissue; cartilage, 152 ± 46 ng/g tissue) (Figure 2B). Interestingly, the release of visfatin/Nampt was significantly higher with synovial membrane than cartilage (*P* = 0.0003) or subchondral bone (*P* = 0.0012), with no difference between OA cartilage and subchondral bone (*P* = 0.08).

**Figure 3:**
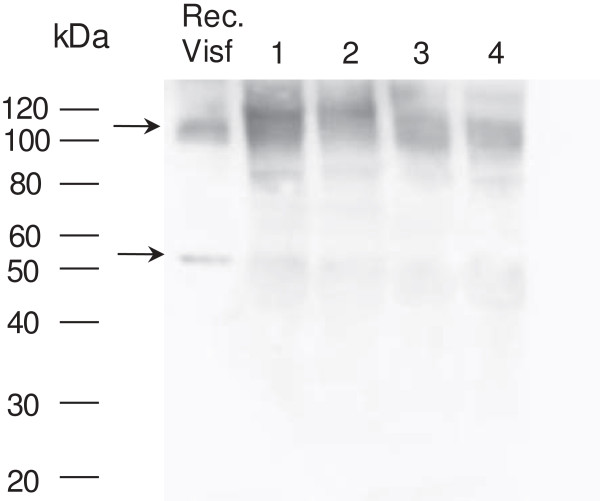
**Presence of visfatin/Nampt in synovial fluid.** Synovial fluids were obtained from four osteoarthritis patients with joint effusion. Western blot analysis of visfatin/nicotinamide phosphoribosyltransferase (Nampt) protein level under nondenaturing conditions, showing polymeric conformation. Arrows show bands specific to visfatin/Nampt.

Since visfatin/Nampt was found to be mainly produced by the synovium, we investigated the presence of the protein in OA synovial fluid. Again, under nondenaturing conditions, visfatin/Nampt protein was present in the dimeric conformation in synovial fluid (Figure 3).

**Figure 4:**
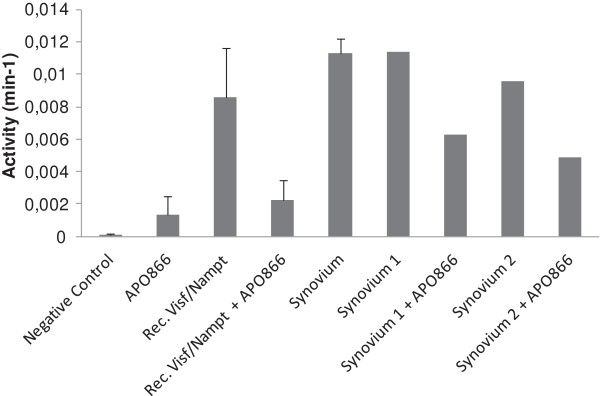
**Enzymatic activity of visfatin/Nampt in synovial membrane of human osteoarthritis.** Enzymatic activity of visfatin/nicotinamide phosphoribosyltransferase (Nampt) assayed in protein extracts of human osteoarthritis synovial membranes by colorimetric assay. Absorbance at 450 nm was measured every 5 minutes for 1 hour, representing the appearance of Nampt product, nicotinamide mononucleotide, over time. Negative control, water; positive control, recombinant visfatin/Nampt (50 μg/ml). Data are mean ± standard error of the mean of 3 samples. In parallel, recombinant visfatin/Nampt and two synovium samples were pretreated for 1 hour at 37°C with APO866 (10 nM). Histograms show the enzymatic Nampt activity displayed by the sample (per minute), calculated from the slope of the straight line of the curve, which is obtained by representing the absorbance (optical density) over time (per minute).

Considering the synovial membrane as the most productive tissue of visfatin/Nampt within the OA joint, we assessed the enzymatic activity of visfatin/Nampt within the synovium. By calculating the slope of the straight curve corresponding to the production of nicotinamide mononucleotide over time, we identified a detectable Nampt activity in the synovium of three patients that was in the same range as the recombinant visfatin/Nampt (Figure 4). The use of the specific inhibitor APO866 decreased this enzymatic activity for the recombinant visfatin/Nampt and for the two synovial membranes. The inhibition for the synovial samples may be less effective in this assay since the kit has been designed for purified protein but not for crude extracts.

### Enzymatic visfatin/Nampt is involved in the proinflammatory activation of murine chondrocytes

**Figure 5:**
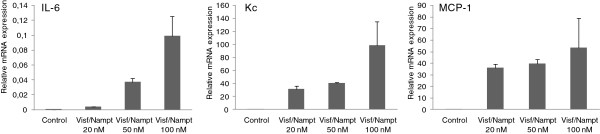
**Dose–response effect of visfatin/Nampt on the mRNA expression of interleukin-6 (IL-6), keratinocyte chemoattractant (Kc) and monocyte chemoattractant protein 1 (MCP-1) by murine chondrocytes (from left to right).** Murine chondrocytes were treated with recombinant visfatin/nicotinamide phosphoribosyltransferase (Nampt) at 20, 50 and 100 nM for 24 hours. Quantitative reverse transcriptase-polymerase chain reaction analysis of mRNA levels relative to that of murine hypoxanthine–guanine phosphoribosyltransferase. Data are mean ± standard error of the mean of three experiments.

**Figure 6:**
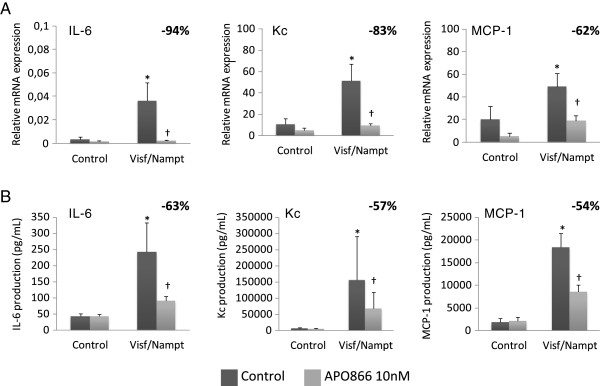
**Proinflammatory effect of visfatin/Nampt and effect of its enzymatic blockade by APO866 on murine chondrocytes.** Murine chondrocytes were pretreated or not with 10 nM APO866 for 4 hours, and then with 100 nM recombinant visfatin/nicotinamide phosphoribosyltransferase (Nampt). **(A)** Quantitative reverse transcriptase-polymerase chain reaction analysis of mRNA levels of interleukin (IL)-6, keratinocyte chemoattractant (Kc) and monocyte chemoattractant protein 1 (MCP-1) relative to that of hypoxanthine–guanine phosphoribosyltransferase, *n* = 6. **(B)** Enzyme-linked immunosorbent assay of protein release of IL-6, Kc and MCP-1, *n* = 6. *P < 0.05 versus nonstimulated control; †*P* < 0.05 versus visfatin/Nampt alone. The percentage corresponds to the average decrease of mRNA or protein level with APO866 pretreatment. Data are mean ± standard error of the mean of six experiments.

Because visfatin/Nampt was found in the dimeric form *ex vivo* in human OA joints, we investigated the effect of its enzymatic activity on chondrocyte activation. Murine chondrocytes were stimulated with increasing concentrations of visfatin/Nampt (20, 50 and 100 nM). We measured the mRNA expression of three major cytokines involved in OA pathophysiologic features, IL-6, Kc and MCP-1, to investigate visfatin/Nampt proinflammatory effects. Visfatin/Nampt at the different doses stimulated the mRNA expression of all three cytokines, with maximal effect at 100 nM visfatin/Nampt (Figure 5). We chose 100 nM visfatin/Nampt for further experiments because it produced the maximal increase in mRNA expression. The visfatin/Nampt-mediated increase in mRNA levels for IL-6, Kc and MCP-1 was 10.6-fold (*P* = 0.02), 4.9-fold (*P* = 0.02) and 2.5-fold (*P* = 0.03), respectively, compared with controls (Figure 6A) and was associated with an increase in protein levels (*P* = 0.02) (5.8-fold, 29.7-fold and 10.5-fold, respectively, compared with unstimulated controls) (Figure 6B). In contrast, visfatin/Nampt did not stimulate vascular endothelial growth factor and transforming growth factor beta nor the hypertrophic differentiation markers Indian hedgehog, type X collagen and runt-related transcription factor 2 (data not shown).

To determine the involvement of Nampt enzymatic activity in these proinflammatory cytokinic effects, chondrocytes were pretreated with APO866 (10 nM) for 4 hours before visfatin/Nampt stimulation (100 nM). This dose of APO866 was selected from our previous work [28]. APO866 significantly decreased the mRNA expression of IL-6, Kc and MCP-1 (*P* = 0.02) (inhibitory effect of 94%, 83% and 62%, respectively, compared with visfatin/Nampt alone; Figure 6A) and protein levels (inhibition of 63%, 57% and 54% for IL-6, *P* = 0.02; Kc, *P* = 0.03; and MCP-1, *P* = 0.02, respectively, compared with visfatin/Nampt alone; Figure 6B).

### The enzymatic activity of visfatin/Nampt is involved in the proinflammatory activation of murine osteoblasts

**Figure 7:**
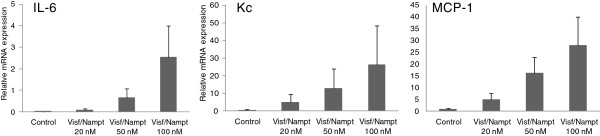
**Dose–response effect of visfatin/Nampt on the mRNA expression of interleukin-6, keratinocyte chemoattractant and monocyte chemoattractant protein 1 by murine osteoblasts.** Murine osteoblasts were treated with recombinant visfatin/nicotinamide phosphoribosyltransferase (Nampt) at 20, 50 and 100 nM for 24 hours. Quantitative reverse transcriptase-polymerase chain reaction analysis of mRNA levels of (left to right) interleukin-6, keratinocyte chemoattractant and monocyte chemoattractant protein 1 relative to that of hypoxanthine–guanine phosphoribosyltransferase. Data are mean ± standard error of the mean of four experiments.

**Figure 8:**
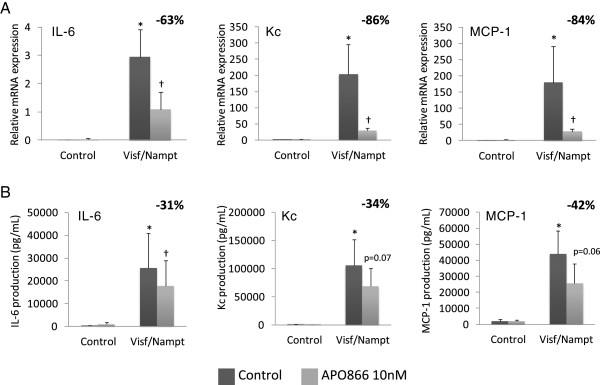
**Proinflammatory effect of visfatin/Nampt and effect of its enzymatic blockade by APO866 on murine osteoblasts.** Murine osteoblasts were pretreated or not with 10 nM APO866 for 4 hours, and then with 100 nM recombinant visfatin/nicotinamide phosphoribosyltransferase (Nampt). **(A)** Quantitative reverse transcriptase-polymerase chain reaction analysis of mRNA levels of interleukin (IL)-6, keratinocyte chemoattractant (Kc) and monocyte chemoattractant protein 1 (MCP-1) relative to that of hypoxanthine–guanine phosphoribosyltransferase, *n* = 6. **(B)** Enzyme-linked immunosorbent assay of protein release of IL-6, Kc and MCP-1, *n* = 6. *P < 0.05 versus nonstimulated control; †*P* < 0.05 versus visfatin/Nampt alone. The percentage corresponds to the average decrease of mRNA or protein level with APO866 pretreatment. Data are mean ± standard error of the mean of six experiments.

To study the effect of visfatin/Nampt on osteoblasts, we conducted similar experiments to those performed with chondrocytes. Osteoblasts were stimulated with increasing doses of recombinant visfatin/Nampt (20, 50 or 100 nM) and the expression of IL-6, Kc and MCP-1 was measured (Figure 7). The concentration of 100 nM was the most efficient in stimulating osteoblasts for all mediators (*P* = 0.06) and was thus chosen for all subsequent experiments with osteoblasts. Visfatin/Nampt significantly increased the mRNA expression of IL-6, Kc and MCP-1 (296-fold, *P* = 0.03; 262-fold, *P* = 0.02; and 161-fold, *P* = 0.02, respectively, compared with the control; Figure 8A) and protein levels (increase of 115-fold, *P* = 0.02; 164-fold, *P* = 0.02; and 22-fold, *P* = 0.03, respectively, compared with the control; Figure 8B). Again, visfatin/Nampt did not modify the expression of vascular endothelial growth factor and transforming growth factor beta in osteoblasts (data not shown).

Before characterizing the role of the enzymatic activity of visfatin/Nampt on osteoblasts, we determined the most efficient dose of APO866 for Nampt blockade. Osteoblasts were pretreated for 4 hours with 1, 10 or 100 nM APO866 before stimulation with 100 nM visfatin/Nampt, which could decrease the expression of IL-6, Kc and MCP-1 (data not shown). APO866 at 10 and 100 nM was the most efficient and we used the same concentration of APO866 as for chondrocytes (10 nM). A cytotoxicity test measuring lactate dehydrogenase activity at 10 and 100 nM APO866 showed no severe mortality (90% survival; data not shown).

Pretreating osteoblasts with APO866 thus significantly decreased visfatin/Nampt-induced mRNA levels of IL-6, Kc and MCP-1 (63% inhibition, *P* = 0.03; 86% inhibition, *P* = 0.02; and 84% inhibition, *P* = 0.04, respectively, compared with visfatin/Nampt alone; Figure 8A) and protein levels (31% inhibition, *P* = 0.03; 35% inhibition, *P* = 0.07; and 42% inhibition, *P* = 0.06, respectively, compared with visfatin/Nampt alone; Figure 8B).

## Discussion

In the present study we show that visfatin/Nampt is produced by the three main tissues of the human OA joint but to a greater degree by synovium. Visfatin/Nampt is naturally produced in a dimeric conformation by OA tissues, which corresponds to its enzymatically active form. Moreover, Nampt enzymatic activity is involved in the proinflammatory effects of visfatin/Nampt on chondrocytes and osteoblasts.

The detection of visfatin/Nampt in OA tissues has been reported previously, showing that all OA human tissues expressed visfatin/Nampt (that is, cartilage, subchondral bone, synovium as well as infrapatellar fat pad), but the conformation was not assessed [36,37]. Interestingly, Meier and colleagues detected visfatin/Nampt by immunohistochemistry within OA synovium, especially around vessels [26]. Here, we describe the conformation of visfatin/Nampt produced by the joint and its enzymatic activity. We previously reported that cultured human OA chondrocytes express visfatin/Nampt in response to IL-1β [17]. Here, we demonstrate that the three main tissues of the human OA joint (that is, cartilage, subchondral bone and synovial membrane) store and release visfatin/Nampt, which is more significantly produced by synovial membrane than cartilage and subchondral bone. This finding suggests a potential paracrine effect of visfatin/Nampt from synovium to the other tissular and cellular components of the joint. Similarly, other mediators such as TNFα or IL-1β are released by the action of OA-activated synovial membrane on adjacent cartilage and subchondral bone tissues [2]. In agreement with Duan and colleagues, visfatin/Nampt was present in synovial fluid from OA patients [38].

Visfatin/Nampt is a unique proinflammatory adipokine because it displays both cytokinic and enzymatic activities, the latter requiring a dimerization of two 52 kDa monomers to organize the enzymatic active site capable of converting nicotinamide to nicotinamide mononucleotide [19,39]. We therefore investigated whether these dual roles of visfatin/Nampt are linked and are involved in OA pathophysiologic features. Use of nondenaturing conditions revealed that visfatin/Nampt stored and released by OA human tissues is naturally dimerized, because we detected the 120 kDa form. This natural dimeric form has been detected in human serum and is also constitutively released by human hepatocytes [40,41].

Interestingly, visfatin/Nampt is also released by infrapatellar fat pad: the role of such a release by this tissue needs to be further addressed considering the intracapsular but extrasynovial localization of this tissue [37].

Because visfatin/Nampt came predominantly from the synovium, we assessed and found that it had synovial enzymatic activity. We did not systematically find Nampt activity in other OA joint tissues, probably because of the lower amount of visfatin/Nampt stored in these tissues (data not shown). We were not able to measure the enzymatic activity in CM or synovial fluid because visfatin/Nampt is much more diluted there than in tissues. Furthermore, we could not discriminate which cell type (that is, fibroblastic synoviocytes or infiltrating mononuclear cells) showed Nampt activity in OA synovium. Given the increased synovial production of visfatin/Nampt in an enzymatically active form, we hypothesize that visfatin/Nampt present in the OA joint originates mainly from synovium and acts on adjacent cartilage and subchondral bone in a paracrine pathway, with its enzymatic activity involved in its cytokinic effect.

The effect of visfatin/Nampt on synovial fibroblasts from patients with rheumatoid arthritis has been extensively studied and is characterized by proinflammatory and prodegradative effects (that is, IL-6, IL-8, MCP-1 and MMP release) [16,26,27]. Here, we investigated the effects of visfatin/Nampt on chondrocytes and, for the first time, on osteoblasts. Our team had demonstrated that human OA chondrocytes stimulated by visfatin/Nampt could acquire a prodegradative and proinflammatory phenotype by increasing prostaglandin E_2_, MMP-3 and MMP-13 [17] Here, we further characterize the visfatin/Nampt-induced proinflammatory phenotype of chondrocytes since visfatin/Nampt was also responsible for stimulation of a cytokine (IL-6) and chemokines (Kc, MCP-1), all mediators critical in the OA pathological process because they participate in cartilage extracellular matrix degradation and attraction of proinflammatory cells [2,3]. Despite the ubiquitous proinflammatory effect of visfatin and considering the ubiquitous role of Nampt enzymatic activity, such effects remain selective because we did not find any change in expression of growth factors or hypertrophic differentiation markers. Busso and colleagues treated efficiently collagen-induced arthritic mice with the specific enzymatic inhibitor APO866 and found decreased expression of proinflammatory mediators (IL-1β, IL-6 and MCP-1) but not other mediators (IL-10, interferon-gamma, regulated upon activation normal T-cell expressed and presumably secreted [RANTES], and IL-12), which again illustrates the selective inhibitory effect of APO866 [18].

To determine the involvement of the enzymatic activity of visfatin/Nampt in these proinflammatory effects, we pretreated chondrocytes and osteoblasts with the inhibitor APO866 – which specifically antagonizes the enzymatic activity only if the visfatin/Nampt dimerizes. The induction of the proinflammatory cytokines was greatly decreased (up to 94% and 63% for IL-6 in chondrocytes and osteoblasts, respectively), which demonstrates that the proinflammatory effects of visfatin/Nampt on chondrocytes greatly depend on its enzymatic activity. In agreement, we previously reported a similar decrease in visfatin/Nampt-induced prostaglandin E_2_ release with APO866 treatment [28]. The inhibitory role of APO866 seems to specifically antagonize the proinflammatory effects of recombinant visfatin/Nampt and is not due to depletion of NAD (resulting in slower cellular function) or to inhibition of endogenous intracellular visfatin/Nampt. Indeed, we stimulated chondrocytes with IL-1β (0.1 ng/ml) treated with APO866 (10 nM) and found no decrease in mRNA levels of IL-6, Kc or MCP-1 (data not shown) while IL-1β is known to induce intracellular visfatin/Nampt in chondrocytes [17]. In other conditions and other cell types, extracellular visfatin/Nampt had similar effects because it induced proinflammatory signaling in human vascular smooth muscle cells through Nampt activity, which was blocked by APO866 treatment [42].

We investigated the effects of visfatin/Nampt on murine osteoblasts for the first time. Osteoblasts were sensitive to visfatin/Nampt because their stimulation induced the expression and production of the same proinflammatory cytokines as in the chondrocyte experiments (that is, IL-6, Kc and MCP-1). Interestingly, the induction of proinflammatory cytokines was much higher in osteoblasts than chondrocytes. As seen with chondrocytes, the use of APO866 on osteoblasts decreased the production of proinflammatory cytokines, which confirms the enzymatic effect of visfatin/Nampt on osteoblasts. Visfatin/Nampt has a proinflammatory effect on the three main cell types within the joint: chondrocytes, osteoblasts (depending in part on Nampt activity) and synoviocytes [16,26,27].

## Conclusion

Visfatin/Nampt is produced and stored by all three major tissues of the human OA joint, mainly synovial membrane, under a dimeric conformation necessary to locally exert its enzymatic action. Since this adipokine may activate chondrocytes but also osteoblasts and acts mainly by modulating NAD synthesis, targeting specifically this Nampt enzymatic activity with the oral compound APO866 may open new therapeutic perspectives in OA.

## Abbreviations

CM: conditioned media; DMEM: Dulbecco’s modified Eagle’s medium; ELISA: enzyme-linked immunosorbent assay; IL: interleukin; Kc: keratinocyte chemoattractant; MCP-1: monocyte chemoattractant protein 1; MMP: matrix metalloproteinase; NAD: nicotinamide adenine dinucleotide; Nampt: nicotinamide phosphoribosyltransferase; TNFα: tumor necrosis factor alpha.

## Competing interests

The authors declare that they have no competing interests.

## Authors’ contributions

M-CL, XH, CB, CJ, FB and JS were responsible for the study design, manuscript preparation, and interpretation of the data. AS and GN carried out all human sample collection, and participated in the study design of experiments using human samples, in the interpretation of the data and revising the manuscript. M-CL performed the experiments. CB and MG contributed to the *in vitro* experiments on murine cells. AS, GN, XH, JS and FB were responsible for collection of human OA tissues and synovial fluid samples. All authors reviewed and approved the final manuscript.

## Supplementary Material

Additional file 1: Table S1 Presenting specific mouse primer sequences. HPRT, hypoxanthine–guanine phosphoribosyltransferase; Ihh, Indian hedgehog; Runx2, runt-related transcription factor 2; TGFβ, transforming growth factor beta; VEGF, vascular endothelial growth factor. Click here for file
