# Supplementary material for: Expression and function of visfatin (Nampt), an adipokine-enzyme involved in inflammatory pathways of osteoarthritis
Source: Arthritis Res Ther. 2014 Jan 31;16(1):R38. doi: 10.1186/ar4467 (PMC3978827; doi:10.1186/ar4467)
Supplement: Additional file 1: Table S1 — Presenting specific mouse primer sequences. HPRT, hypoxanthine–guanine phosphoribosyltransferase; Ihh, Indian hedgehog; Runx2, runt-related transcription factor 2; TGFβ, transforming growth factor beta; VEGF, vascular endothelial growth factor. [file ar4467-S1.doc]

Additional file 1: Table S1. Specific mouse primer sequences

|  | **Forward** | **Reverse** |
| --- | --- | --- |
| **HPRT** | 5’-AGGACCTCTCGAAGTGT-3’ | 5’-ATTCAAATCCCTGAAGTACTCAT-3’ |
| **IL-6** | 5’-GTCACAGAAGGAGTGGCTA-3’ | 5’-AGAGAACAACATAAGTCAGATACC-3’ |
| **Kc** | 5’-GGGCCAAGAGAATATCCGA-3’ | 5’-TAGGGTTGCCAGATTTAACAG-3’ |
| **MCP-1** | 5’-GATGATCCCAATGAGTAGGCT-3’ | 5’-TTCTGATCTCATTTGGTTCCGA-3’ |
| **IL-1β** | 5’-GGGCCTCAAAGGAAAGAATC-3’ | 5’-CCACTTTGCTCTTGACTTCTATC-3’ |
| **VEGF** | 5’-CTGCTGTGGACTTGTGTTG-3’ | 5’-ATGGGTTTGTCGTGTTTCT-3’ |
| **TGFβ** | 5’-GCGGTGCTCGCTTTGTA-3’ | 5’-CTGTGTGAGATGTCTTTGGTT-3’ |
| **Ihh** | 5’-CACCTTCAGTGATGTGCTTATTT-3’ | 5’-GGGCTGCTGGTTCTGTA-3’ |
| **Type X Collagen** | 5’-GATGCCGCTTGTCAGTG-3’ | 5’-TAAAGATACCAGATCTTGGGTCG-3’ |
| **Runx2** | 5’-ATTCATCCTGACTCCCTCT-3’ | 5’-CTACCACACACAAACAATAAATAGC-3’ |
